# How recovery influences community reintegration: perspectives of persons with spinal cord injury and their support persons

**DOI:** 10.3389/fresc.2025.1617764

**Published:** 2025-09-11

**Authors:** Anne M. Bryden, Brian Gran, Susan Hinze, Mary Ann Richmond, Kim D. Anderson

**Affiliations:** ^1^ The MetroHealth Center for Rehabilitation Research, The MetroHealth System, Cleveland, OH, United States; ^2^ Department of Physical Medicine and Rehabilitation, Case Western Reserve University School of Medicine, Cleveland, OH, United States; ^3^ Department of Sociology, Case Western Reserve University, Cleveland, OH, United States; ^4^ Spinal Cord Injury/Disorders Center, Veterans Affairs Northeast Ohio Healthcare System, Cleveland, OH, United States

**Keywords:** spinal cord injury, community reintegration, recovery, participation, caregiver

## Abstract

**Purpose:**

To investigate how perceived recovery influences perspectives on successful community reintegration, from the point of view of persons with spinal cord injury (PWS) and their support persons (SP).

**Methods:**

Our mixed methods approach included qualitative interviews conducted with civilians and Veterans with spinal cord injury (SCI) and their designated SP at three time points across the first year after SCI: during inpatient rehabilitation, 6 months, and 12 months. Participants with SCI completed measures of independence [Spinal Cord Injury Independence Measure III (SCIM-III)] and self-efficacy (Moorong Self-Efficacy Scale) during inpatient rehabilitation and at 12 months postinjury. Data analysis was informed by the Transformative Framework and International Classification of Functioning, Disability, and Health (ICF).

**Results:**

Regarding perceptions of how recovery influences community reintegration, PWS most often reported themes related to slow recovery whereas SPs expressed concerns about psychological impacts on PWS. While some participants were equally satisfied with rate of recovery and rate of community reintegration, several deviated from that expected trajectory. Associations between satisfaction with community reintegration and independence or self-efficacy were variable.

**Conclusions:**

Successful community reintegration cannot be predicted solely on clinical measures. Inclusion of perspectives of PWS and their support systems is critical to inform successful societal participation after SCI.

## Introduction

Following spinal cord injury (SCI), people experience life-changing physical and emotional impacts. Community reintegration (CR) is the ultimate goal of rehabilitation for people who experience these injuries. Community reintegration is a broad term that encompasses many aspects of life participation, including access to housing, moving around one's community, participating in work, educational and leisure activities, and engaging in meaningful social relationships and community roles (1). Anderson et al. (2) were among the first to investigate definitions of community reintegration from the perspectives of persons with spinal cord injury (PWS) and their support persons (SP), demonstrating priorities of returning to social activities and roles, being active by participating in or pursuing employment, establishing independence, and emotionally adjusting to a new life.

Unfortunately, PWS experience challenges to successful community reintegration due to increasingly shortened inpatient rehabilitation lengths of stay and subsequent difficulty accessing sufficient and appropriate resources in the community (3). Not surprisingly, interventions to facilitate community reintegration have been included in rehabilitation programs. In a scoping review, Sulaiman et al. (4) investigated the characteristics of existing community reintegration intervention programs to identify their content, strengths, and limitations, and reported effectiveness, finding a predominance of behavioral interventions, transitional rehabilitation programs, and peer mentoring. While most studies demonstrated effectiveness in critical community reintegration domains, effects were not always sustained at longer-term follow-up. In addition, most of the interventions focused on a specific aspect of community reintegration, which may not be sufficient for individuals with complex needs (4). This introduces the question of how recovery is associated with community reintegration—whether greater recovery begets more successful community reintegration. In an integrative review of SCI heterogeneity on rehabilitation outcomes, Gupta et al. (5) found that while there is evidence that social support and self-esteem have positive effects, and psychological or medical complications have negative effects, on community reintegration, there is a lack of clarity about how injury-related characteristics influence community reintegration.

The purpose of this study is to investigate how perceived recovery influences perspectives on successful community reintegration, from the point of view of both PWS and their SPs. Our qualitative approach, informed by the Transformative Framework (6, 7) and International Classification of Functioning, Disability, and Health (ICF) (8) provides contextual information about reintegration experiences and gives voice to PWS and their SPs who are often marginalized by disability experiences. The ICF is a framework well known to health professionals and used as a tool to structure interventions. The Transformative Framework, however, is not widely used in the physical medicine and rehabilitation community, although it offers value in recognizing power imbalances between people and institutions. Our qualitative approach elicits direct insights about the lived experiences of PWS and SPs while reintegrating into their communities after a severe injury.

## Materials and methods

### Design

Data presented were collected as part of a large qualitative study (2). This longitudinal study used a multimethods approach, combining semistructured interviews and validated outcome measures. The in-depth qualitative interviews explored participants' personal definitions, perceptions, and expectations of recovery and community reintegration. These interviews covered various aspects, including physical feelings, emotional responses, social support, and navigating resources. Participants also discussed barriers and facilitators in their decisions about recovery and reintegration options, and how their recovery experiences affected their reintegration into the community. This article focuses specifically on the findings related to how participants’ perceptions of their recovery influence their options for reintegrating into society*.* Ethics approval was obtained by the MetroHealth Institutional Review Board (IRB19-00323) and the VA Northeast Ohio Healthcare System Institutional Review Board (CY19-033).

### Participants

Participants were recruited using criterion-based purposive sampling, a non-probability technique where investigators select individuals based on specific criteria (9). Eligible participants included individuals aged 18 or older with newly acquired SCI of any cause, level, or severity during their initial inpatient rehabilitation, or their designated support person. We aimed for a diverse sample, striving to enroll equal numbers of individuals with tetraplegia and paraplegia, across all American Spinal Injury Association (ASIA) Impairment Scale (AIS) grades (A–D), various ages, and sexes. Participants with SCI and their support persons were approached in person by the study coordinator during their inpatient SCI rehabilitation stay at either a Veterans Affairs SCI Hub or an academic medical center in the Midwest United States. The enrollment goal was 15 individuals with SCI and 15 support persons at each setting, totaling 60 participants. All participants participated in the informed consent process with the primary investigator and agreed to participate in the study.

### Semistructured interviews

Each participant engaged in three in-depth, semistructured interviews within the first-year post injury: one during inpatient rehabilitation, another around 6 months postinjury (mpi), and a final one at 12 mpi. An interview guide ensured consistent data collection across participants. The open-ended questions, informed by the International Classification of Functioning, Disability, and Health (ICF) and Transformative Frameworks, explored various facets of recovery and community reintegration. Interviews with PWS and their support persons (SP) were conducted separately and lasted 30–60 min. The interview team consisted of four authors (KA, AB, BG, and SH). Interviews were conducted by two of the team members at a time. Once assigned to each PWS-SP pair, the interview team stayed consistent and conducted all three interviews with that dyad.

### Outcome measures

To complement the semistructured interviews, we collected three validated outcome measures for the PWS. Data from the International Spinal Cord Injury (SCI) Core Dataset version 2.0 (10), including length of hospitalization, cause, level, and severity of injury, and discharge location, were obtained through a chart review of the inpatient rehabilitation stay. Concurrently with the inpatient and 12-month interviews, the Spinal Cord Independence Measure version III (SCIM III) (11) and the Moorong Self-Efficacy Scale (MSES) (12) questionnaires were administered via interview to assess broad changes in functional independence and self-efficacy over the first 12 mpi. SCI characteristics, and SCIM III and MSES data can be found in Anderson et al. (2).

### Data analysis

Interviews were recorded using a digital audio recorder. A professional transcription service (Rev.com, San Francisco, CA, USA) transcribed each recording verbatim. Study staff then reviewed these transcripts for accuracy and removed identifying information. The deidentified transcripts were organized and analyzed using NVivo data management software. Initially, four authors (KA, AB, BG, and SH) independently coded the transcripts. Subsequently, the team reached a consensus on the codes through group review sessions. Themes and subthemes were developed using a constructivist grounded theory analytic approach (13) until theoretical saturation was achieved (14). This critical inquiry method recognizes the researcher's active role in constructing, shaping, and interpreting data and emphasizes deep reflexivity to examine assumptions, biases, and preconceptions throughout the research process (15). To ensure data credibility, we conducted verification checks and obtained feedback through multiple discussions with our community partner, the local chapter of the United Spinal Association.

Among several topics in the interview guide, PWS and SPs were asked to describe their satisfaction with their rate of recovery (or their loved one's rate of recovery) over the past year. Responses to this question were analyzed and assigned a satisfaction with recovery score based on a three-point scale: 1 = low satisfaction with recovery, 2 = medium satisfaction with recovery, and 3 = high satisfaction with recovery. Similarly, PWS and SP participants’ responses to how satisfied they were with their rate of community reintegration (or that of their loved one) at 1 year were assigned a satisfaction score based on the following three-point scale: 1 = low satisfaction with CR, 2 = medium satisfaction with CR, and 3 = high satisfaction with CR. All four authors who conducted the interviews assigned scores, and any disagreements were resolved through discussion. Data trustworthiness was established via multiple approaches (37, 38). First, credibility of data was ensured through three interviews conducted over 1 year, diversity of participants, and by having multiple analysts, independent analyses, and regular team meetings to examine assumptions, biases, and preconceptions throughout the study. Member checking was conducted during the interview process by summarizing content back to each interviewee and asking for clarification. In addition, analyses were reviewed with the non-interviewing author (MAR) for interpretation, as well as with our community partner, a local chapter of the United Spinal Association, for feedback.

Changes in independence (SCIM-III) and self-efficacy (MSES) were analyzed over the first year after injury, and we used the following information to inform our analyses. According to Scivoletto et al. (16), changes in SCIM-III scores of four points or more indicate a small clinically significant improvement, whereas changes of 10 points or more demonstrate a substantial improvement. However, interpretations should be made cautiously, as scholars have argued that they rely on parametric statistical assumptions that may not have been met, and do not include the informed opinions of clinical investigators or participants with SCI (17). The literature shows that clinically meaningful changes in self-efficacy have been determined to be four points or greater (18), and that scores of 80 or higher indicate reasonable adjustment to SCI (19).

## Results

### Participants

Forty-four participants (civilians and Veteran PWS and their designated SPs) were enrolled from January 2020 through June 2022. The cohort included 23 PWS (16 civilians and seven Veterans) and 21 SPs (16 SPs of civilians and five SPs of Veterans). While regulatory restrictions at the Veterans Affairs Hospital due to the COVID-19 pandemic limited meeting our targeted enrollment of Veteran PWS and SPs, theoretical saturation was reached. Detailed demographic information can be found in Anderson et al. (2); the cohort of PWS was predominantly male (19), predominantly tetraplegic (14), and were classified as AIS A (six), AIS B (5), AIS C (5), and AIS D (7). Veterans were slightly older, with a mean age of 52 years compared to civilians’ mean age of 41 years. Average inpatient rehabilitation lengths of stay were 36 days (range 15–49) for civilian PWS and 60 days (range 43–80) for Veteran PWS.

### Recovery influence on community reintegration

#### Persons with spinal cord injury

At the second and/or third interview time points (6 and 12 mpi) PWS were asked to describe how they felt their recovery influenced success at getting back into life. Table 1 presents representative quotes for the main themes constructed from the data (civilians *n* = 13, Veterans *n* = 7). The most common theme from civilian PWS related to slow recovery or recovery taking time, whereas the most common theme from the Veteran PWS was how physical recovery or functional abilities contributed to community reintegration. For these Veterans, in all cases but one, participants reported that improvements in their recovery or abilities had a positive effect on community reintegration. One participant identified that a lack of recovery negatively affected community reintegration. Two themes constructed from the civilian data that were not reported by the Veterans related to COVID-19 as a barrier and thinking about returning to work. Conversely, two themes constructed from the Veteran data that were not reported by civilians included weather as a barrier and poor knowledge about SCI.

**Table 1:** Recovery influence on reintegration—people with SCI (themes and representative quotes).

Theme	Civilian PWS	Veteran PWS
Time/slow recovery	*Well, uh, I, I feel like, uh…I, I feel my recovery is extremely slow. Uh, and it, it, it doesn't, it doesn't contribute to anything, quite frankly.* (C-PWS-4, 12 months) *Slow process. Um, everything has gone fine, it's just been a slow process.* (C-PWS-12, 12 months)	*So that's probably the best I can put it, you know? I, I'm, I'm recovering today and a year from now, I'll still be recovering, right? I'm, I'm never going to be finished recovering.* (V-PWS-8, 6 months) *Hmm. It just seems slow. To me. Um, but that might be part of my fault, too, so I don't know. Researcher: Your fault in what way? 107: Um, just not doing more, I guess.* (V-PWS-7, 12 months)
Psychological/emotional state	*The current recovery has seen ups and downs. Like, some days I feel strong and I can handle this. Other days, not so much. So, it varies day by day.* (C-PWS-2, 6 months) *Um, my recovery time, you know, it's kinda hard to say, I mean I know I'm doing better every day, everything I do makes me better. But it's just hard for me to, uh, you know psychologically deal with this.* (C-PWS-20, 12 months)	*You know, you got to be determined to…To go through this, you got to be determined. You can't go in it half…You know, and expect good results, and if you're just gonna lay in bed and stuff, that's what's gonna happen. You're gonna end up laying in bed, you know?* (V-PWS-8, 12 months) *It's learning to deal with my limitations, I'm confronted every day with a different aspect of what I cannot do, that I'm familiar with doing on an everyday basis, or used to be… So, learning to live within my limitations is one of the greatest eye openers that I have. Both positive and negative.* (V-PWS-1, 6 months)
Physical or functional recovery	*It's like, just my little leg movement that I've gotten back has, you know, giving me the confidence that, Hey, you know, everything is going to be okay at some point and its making me want to go out and do stuff more and you know.* (C-PWS-22, 6 months) *It is…Because I didn't think I would get any recovery, to watch it come back every day and getting a little bit better and a little bit better, a little bit better. It kind of…you can see that there is a means to an end. So, um, I'm certainly…yeah, I think the recovery has been very beneficial in getting better.* (C-PWS-10, 12 months)	*I mean, it's allowed me to get back into life, um, pretty well. There's been a few strug- like I said, there- there's been a few struggles as far as just not being able to use my other hand. But re- recovery's not, recovery's far from over, so.* (V-PWS-13, 6 months) *Uh, shoot. I'd say they're about equal. The farther along I am in recovery, the more I'm able- the more successful I am about getting back out into the world.* (V-PWS-15, 12 months)
Therapy influence	*I mean, I'm doing what, I'm doing pretty much what I can do here at home. So that's fine, you know, from therapy some where's later on that, that’s going to help me. But I don't know about that. Not everybody, not every therapy outfit can do, give me what I actually need.* (C-PWS-17, 6 months) *Just like the positive you know, stuff that they say when I'm in therapy saying they've noticed improvements and stuff, I think that's helping me drive to work harder and become more successful when it comes to my recovery.* (C-PWS-22, 6 months)	*I try to walk and everything, but, a hour- hour, a- a- a hour a week of therapy in here and, uh, when I stand on my feet, it's not working. So, I'm, next, pay somebody out my own pocket-…to help me walk or stand.* (V-PWS-6, 6 months)
Access to resources	*Ah, I'm getting access to different things, different people. The Internet's been helping a lot and word of mouth.* (C-PWS-3, 6 months)	*Um, but now, I mean, just slowly, we are putting things in place (home modifications) that allow me to have more freedom and, and that's a huge plus.* (V-PWS-7, 6 months)

#### Support persons

SP were asked to describe how they felt their loved one's recovery influenced their success at getting back into life (6 and/or 12 mpi). Table 2 presents representative quotes for the main themes constructed from the data. A prominent theme from both the civilian and Veteran SP related to the psychological or emotional health of their PWS. In addition, both groups were concerned about physical and functional recovery. Three civilian SP talked about recovery time, or recovery as a slow process, whereas this was mentioned by only one Veteran SP. Additional themes that were mentioned by one or two civilian and Veteran SP included impacts on their routine and weather as a barrier to reintegration. Several themes were mentioned by civilian SP, but not mentioned by Veteran SP. These themes included work/employment, therapy's influence on reintegration, influence of family members and friends, access to resources, and COVID-19 as a barrier.

**Table 2:** Recovery influence on reintegration—support persons (themes and representative quotes).

Theme	Civilian SP	Veteran SP
Psychological/emotional state	*I think his recovery of taking one day at a time allows him mentally to know that, um, there's gonna be barriers where there's emotional, physical barriers of actually back to himself. Um, but it also allows him to know that he may not be himself at a hundred percent, he may have this new normal. Um, so I think he's strong willed, he's a very resilient guy. He's not one to dwell, um, and I consider him mentally and emotionally strong. Um, I- I think that helps him with his day-to-day successes* (C-SP-19, 6 months) *So in six months, maybe three or four times. It's literally just overwhelmed him. And, and it's just an overwhelming thing for, like, just to work himself to through. Like one time he said, “Just let me cry. Just let me be angry, mom. Just let me be angry.” And I guess he's right. He has every right to be angry. And then he gets through it, and he's upbeat and positive and ready to go and try different things.* (C-SP-18, 6 months)	*I think he, he needs to build his confidence back up. And I think that is hindering him from doing more that he would probably be able to do. But, yeah, I think his, his confidence to do things in places on his own really needs to be worked on*. (V-SP-1, 12 months) *We just had to get some lights put in the house and, you know, he- he made a comment like I used to be able to do that and now I can't do that. But he's realizing that he might not be able to do that, but he can do other things. So some, maybe some of our- our, uh, normal jobs that we used to do around the house kind of switched. You know, he does maybe a little more cooking and he might do a little bit more of the laundry that he…He does things that he can do. So I think that is helping him re, you know, reincorporate into, in mainstream life.* (V-SP-7, 6 months)
Physical or functional recovery	*Um, I mean, I feel like his recovery itself is good for him. And it could be a little bit easier. It makes, it just, I worry, and it takes a lot of the pressure off of me worrying about how he's doing, and what he needs done- and his becoming more independent-…than he even was before. It's changed even in the last month. Say, which is, it's, it's progress. And I can't, I can't be upset about progress.* (C-SP-20, 6 months) *It's (recovery) giving him back his confidence. You know, just as people, if we so used to doing something, then we lose it, it makes you feel as if you don't have it, you know? But the therapy is giving him back what he, what he lost, realistically. So, it's like that's- that's building the confidence in him as an individual. You know, like, you didn't totally lose it, you temporarily lost it. And to get it back, you have to work at getting it back. So- that's, like, a double thumbs up.* (C-SP-14, 6 months)	*If he's in this situation now, you know, if he's thinking about, I'm gonna be in (this position) five years from now, you know, that can be kinda depressing. So he's making progress, so he feels that that gives him more incentive.* (V-SP-8, 6 months) *I think now…I- I think we were very stagnant for a long time because his attitude was, “I'm gonna walk again,” and now that he’s accepting that, no.,.I think we've made a- a lot of progress just within the last month or two with just his recovery…looking forward. We kind of were in a standstill for a long time and now we’re actually pushing forward and making progress.* (V-SP-7, 12 months)
Time/slow recovery	*I think because it's been so slow, um, it's been challenging.* (C-SP-4, 12 months) *I don't wanna say the wrong thing. I don't even wanna think of the wrong thing. When it, when he…if he can get halfway recovered on that left side, I might feel a little different than I'm feeling now. But, um, from what I'm seeing I don't see nothing have changed yet. So I don't wanna say, “Yeah, it’ll be well in six months.” I don't know. I don't…I, I can't say.* (C-SP-21, 12 months)	*Uh, it, it has moments. You know, there's…He gets discouraged, which is understandable 100 percent. Um, but it’s…Hm. I can't say ta- take a, its taking a lot longer. I think it's, it's hard because you’re trying to, you're trying to go through, like, school-…and, but you're also trying to figure out, like, who you are now-..as well. So I mean, it’s, it’s been hard for him. It really has.* (V-SP-15, 12 months)
Effects of SP routine	*Can't be gone the whole day. You know? Um, you know, with him like this. Unless I have somebody here.* (C-SP-17, 6 months)	*So, I think at the moment, we’re just- we’re just trying to get our new routine, um, get used to- to everything. Which has been a rollercoaster. (laughing) It has-…definitely been. I'm glad he's home. I wouldn’t change that for the world, but it's definitely…It's been- it's been a lot.* (V-SP-15, 6 months) *Well, it's put a hold, it’s put a hold on a lot of things and something that I needed to do last week, I couldn't go ‘cause like, I couldn't leave him here. So, I mean, I have to change. I can't go places like I used to go. So, I have to adapt to that.* (V-SP-6, 6 months)
Weather as a barrier	*We go out to dinner. Um, like I said, the only thing that's hindered us is the weather-…in a, in a wheelchair. Other than that, it's not been an issue at all.* (C-SP-22, 12 months)	*Um, well, he- he's anxious for spring, also. So he's not (going out in winter)… You know, he should at least get out and- and, uh- out and about and go out on the deck. You know.* (V-SP-8, 12 months)

### Satisfaction with rates of recovery and community reintegration at 12 months

At 12 mpi, all PWS were asked to reflect on how satisfied they were with their rate of recovery as well as their rate of reintegration. SPs reported their satisfaction with the rates of recovery and reintegration for their PWS.

### Persons with spinal cord injury

**Figure 1:**
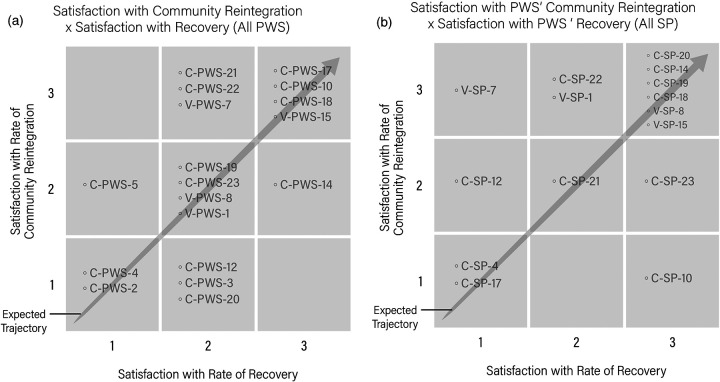
**(a)** Perceived satisfaction with rate of recovery and perceived satisfaction with rate of community reintegration for PWS are compared. The arrow represents an expected trajectory, or congruence in satisfaction between both factors. PWS outside of the expected trajectory represent outliers. **(b)** Support persons’ satisfaction with their PWS’s rate of recovery compared to rate of community reintegration are compared. The arrow represents an expected trajectory, or congruence in satisfaction, between both factors. SPs outside of the expected trajectory represent outliers.

A comparison of perceived satisfaction with rate of recovery and perceived satisfaction with rate of community reintegration shows a moderate correlation (*R* = 0.604), indicating that participants who perceived greater rates of recovery also tended to report greater perceived rates of community reintegration (Figure 1A). Despite this relative alignment, a striking pattern is seen in 10 participants who reported medium satisfaction with their rates of recovery yet more varied levels of satisfaction with rates of community reintegration. We investigated the following exceptions to the expected trajectory: (1) individuals who reported medium levels of satisfaction with rate of recovery but low satisfaction with rate of community reintegration, (2) individuals who reported medium levels of satisfaction with rate of recovery and high satisfaction with rate of community reintegration, (3) individuals with low satisfaction with rate of recovery and medium rate of satisfaction with rate of community reintegration, and (4) individuals with high satisfaction with rate of recovery but medium satisfaction with rate of community reintegration. To better understand contributing factors, we studied the Moorong self-efficacy scores, SCIM-III scores, and qualitative data coded under “recovery influence on community reintegration” and “satisfaction or dissatisfaction with community reintegration” for each of the outliers (Table 3).

**Table 3:** Persons with SCI's satisfaction with rate of recovery and satisfaction with level of community reintegration: outliers from the expected trajectory.

Participant	SCIM-III d/c—1 year	Moorong SES d/c—1 year	Themes	Representative quotes
Medium satisfaction with recovery and low satisfaction with community reintegration				
C-PWS-12 C7 AIS A	53–54	102–87	Recovery is slow COVID-19	Slow recovery is intertwined with restrictions from the pandemic in terms of community reintegration. *Reintegrating into the community is nearly impossible with social distancing and the masks and the limitations of places that are open, so it's, it's pretty difficult right now.*
C-PWS-3 C4 AIS B	18–18	88–63	The whole process	*Uh…Could be better. It's just, it's just the whole process.* Interviewer: *Mm-hmm. Can you tell me more about that?* Participant: *Um…I don’t know how to put it. I don't know how to put it. Next question.*
			COVID-19	*Um, not that good. Uh, I just wish I can get out more, and just be around more…*Acknowledges COVID-19 as partial cause.
C-PWS-20 T11 AIS B	54–83	95–94	Lack of home support	Lack of personal support to do additional exercises at home: *I don't know where I would've been, you know what I'm saying, if I would've had help.*
			Psychological effects of injury	*Oh it's just frustrating because I used to have these things, I used to be able to do right away, and I can't do it no more.*
Medium satisfaction with recovery and high satisfaction with community reintegration				
C-PWS-21 T12 AIS D	55–88	88–84	Positivity	*I just take it day by day, so it, it, it was just, it was a, it’s just a long process. I didn't know it was gonna take this long, but I'm still just going through the process to a full recovery.*
			Family influence	*I'm really satisfied, because my brother, he be in my, in my corner, and my, uh, mother be helping me. So that, that really be helping me.*
C-PWS-22 L1 AIS B	64–72	87–104	Recovery boosts confidence	*I think it's uh, affecting it very well, I would say. It's like, just my little leg movement that I've gotten back has, you know, giving me the confidence that, Hey, you know, everything is going to be okay at some point and its making me want to go out and do stuff more and you know, I, I don't want, I wouldn't feel as, I don't know, it's just given me a little extra confidence boost. I think it's, you know, the small things I notice give me a little bit more confidence.*
			Accessible living space	*(I'm) very satisfied. I was even able to find an apartment that was accessible, so…Well, that's a task, I found out. But…(laughs)*
V-PWS-7 C5 AIS B	45–34	93–85	Keep moving forward	*Uh, I feel, slowly but surely, I'm, I'm starting to be able to do more and more things that I used to do prior to getting injured, which is good. Um, I mean, I'll, of course, I won't be able to get everything back, but, uh- physically and mentally, it's all really helpful. And there are times when you're just, you know, completely helpless, um, frustrated, but, um, it passes, you get over it, and, and you just have to keep moving forward. You don't have an option. Um, but now, I mean, just slowly, we are putting things in place that allow me to have more freedom and, and that's a huge plus.*
			Recovery is slow	At one year: *Hmm. It just seems slow. To me. Um, but that might be part of my fault, too, so I don't know. Um, just not doing more, I guess.*
			Making progress	*I'm still unable to do a lot of things, but I am able to do a lot more today than I was a year ago.*
Low satisfaction with recovery and medium satisfaction with community reintegration				
C-PWS-5 C4 AIS C	24–38	57–80	Positivity	*There's more to go. Yeah. Yeah, there's more to go. But I'm in a good state of mind right now, so, you know, I don't know, I just feel good with everything right now. I'm halfway there.*
High satisfaction with recovery and medium satisfaction with community reintegration				
C-PWS-14 C8 AIS D	55–84	95–103	Being practical	*Uh, I still feel my recovery is, it will definitely need some time, but I am able to do some of the things that I was able to do before. But I know there are still some things that I need help with and that's okay*
			Patience	*Just being a young man who's used to stuff like that, you know, in my 20s, so it's, like, you know, I wanna live on, God, do the things I did before. But, you know, with this whole life change, um, it- it put a stop to things and it did change my life for the better or worse, but it was a major change. So, you know, it was, like, even if I wanted to do this like I did before and do something new, I definitely just have to wait and be more patient.*
			COVID-19	*So it was, like, you know, even if I did become impatient, I just…it's, like, you know, COVIDs still a thing, too, so you're not really missing much out there.*

#### Medium satisfaction with recovery and low satisfaction with community reintegration

Three participants reported medium levels of satisfaction with their rate of recovery but low satisfaction with their rate of community reintegration. Notably, two individuals demonstrated clinically meaningful decreases of self-efficacy by 1 year, while one participant's score remained stable. Interestingly, the SCIM-III scores for the individuals in this outlier group were minimally changed or unchanged for two of the participants over the first year, while the remaining participant experienced an increase by nearly 30 points (Table 3). Qualitatively, participants discussed limitations from the COVID-19 pandemic, slow recovery, lack of support at home, and psychological impacts.

#### Medium satisfaction with recovery and high satisfaction with community reintegration

Three participants reported medium levels of satisfaction with their rate of recovery, and high satisfaction with their rate of community reintegration (Table 3). Each of these individuals had moderate to high self-efficacy scores at 1-year postinjury, one having experienced a 17-point increase within the moderate to high range. Two of the three participants in this outlier group experienced increases in SCIM-III scores of 33 and eight points, while one participant experienced a drop of nine points. Qualitatively, participants reported themes related to positivity, family support, recovery increasing confidence, accessible living spaces, and making progress despite slow recovery.

#### Low satisfaction with recovery and medium satisfaction with community reintegration

One participant reported low satisfaction with rate of recovery and medium satisfaction with rate of community reintegration. The participant experienced a 23-point increase in self-efficacy, crossing from a low to moderate level over the first year after injury, as well as an increase in SCIM-III score over this period. Qualitatively, the participant was positive, acknowledging a good state of mind and more recovery to be had.

#### High satisfaction with recovery and medium satisfaction with community reintegration

The one participant who reported high satisfaction with rate of recovery and medium satisfaction with rate of community reintegration experienced a self-efficacy increase over year one, starting and remaining highly self-efficacious. This individual experienced a nearly 30-point increase in SCIM-III score. Qualitatively, the participant was pragmatic about recovery and function and acknowledged COVID-19 as contributing to his patience with reintegrating into the community.

### Support persons

Comparison of perceived satisfaction with PWS’s rate of recovery and perceived satisfaction with PWS’s rate of community reintegration shows a low correlation (*R* = 0.43), indicating that overall, perceptions were variable and did not follow an expected trajectory of satisfaction with rates of recovery being positively associated with rates of community reintegration (Figure 1B). Those who followed the expected trajectory included six SPs who reported high satisfaction with both their loved one's rate of recovery and rate of community reintegration, one SP who reported medium satisfaction with their PWS’s rates of both recovery and community reintegration, and two SPs who reported low satisfaction for both rates of recovery and community reintegration. We investigated five different scenarios of outliers who deviated from the expected trajectory: (1) SP who reported low level of satisfaction with their PWS's rate of recovery and medium satisfaction with community reintegration; (2) SP who reported a low level of satisfaction with PWS's rate of recovery and high level of satisfaction with community reintegration; (3) SP who reported medium satisfaction with their PWS's rate of recovery and high satisfaction with rate of community reintegration; (4) SP who reported high satisfaction with rate of recovery and low satisfaction with rate of community reintegration; and (5) SP who reported high satisfaction with rate of recovery and medium satisfaction with rate of community reintegration (Table 4).

**Table 4:** Support persons’ satisfaction with rate of recovery and satisfaction with level of community reintegration.

Outlier	Themes	Representative quotes
Low satisfaction with recovery and medium satisfaction with community reintegration (C-SP-12)	Keeping good people around, encouragement	*My view? I just find him to have good people around him that's gonna, you know, build him up with confidence, you know? Not like, make him feel less of a person, you know, be there to build him up, you know, support. That is my biggest thing*. *You know, there's things that you see in his face that, you know, it, it's hard. And I can't say how…You know, I can't sympathize, but I can empathize, you know?…with, with the, the situation. And, um, uh, my biggest thing is just to be the support for him and, you know, give him that, that courage and that, you know, that willpower to keep pushing forward.*
Low satisfaction with recovery and high satisfaction with community reintegration (V-SP-7)	Emotional adjustment to injury	*Um, I think over this last six months it really has, um, increased. I'm pretty happy with it. The first six months, I think he was still kind of in shock. He really didn't wanna leave the house. He was kind of always worried that people were looking at him or, you know, just kind of just apprehensive about going out, and over these last six months, he's- he wants to get out. He wants to go places. He doesn't really care, you know? He's kinda gotten back to his normal, friendly self like, “I'll just say hello to everybody,” and, you know, engage with people.*
Medium satisfaction with recovery and high satisfaction with community reintegration (C-SP-22, V-SP-1)	Recovery boosts optimism/psychological state	*Um I mean I think, the more the recovery is happening and the more successful he is at like therapy and recovery. Then obviously he's gonna be like, in better spirits, too, so then in his everyday life he's gonna be more successful because he's happier.* (C-SP-22) *Um, I'm pretty satisfied with (community reintegration). I mean like I said, like I think he's been successful at doing those things, so therefore, I'm satisfied with them.* (C-SP-22)
	Exceeded initial expectations	*I have been very satisfied. Like I, I did not expect—based on what the neurosurgeon told me, about 54, weeks ago, um, everything has been amazing.* (V-SP-1)
High satisfaction with recovery and low satisfaction with community reintegration (C-SP-10)	Working from home but isolating from community; COVID-19	*Hm. I don't think she's back into life yet though. I think she's back into work, but it, I think it's hard to get back into life right now. We’re…life as we know it, or as she knew it doesn't exist right now. So I, I, I think that's gonna be delayed another year for her because she doesn't want to be around people. She doesn't want to get sick*. *Oh, I'm very dissatisfied. She hasn't got, she has not gotten back into life, period.*
High satisfaction with recovery and medium satisfaction with community reintegration (C-SP-23)	Making progress but not back where he used to be	*Like, that- that kind of implies that I had expectation, you know? And I didn't. I didn't really, you know, and that's kind of how we've been this whole time, just, like, hope for the best and take it as it comes and, you know, celebrate all small victories. So I'm- I'm satisfied enough that I'm so excited to, you know, like, put together clips of all of his (laughs) uh, therapies that I took videos of, and you know, see that progress. I'm- I'm very satisfied with where he's come so far, but I- I just…I want to see him there. You know, I want to see him like I said, you know, in a job and in his truck and- and to be fully himself again.*

#### Low satisfaction with recovery and medium satisfaction with community reintegration

One SP reported low satisfaction with their loved one's recovery and medium satisfaction with their community reintegration, stating the importance of providing encouragement and having a supportive network of friends and family.

#### Low satisfaction with recovery and high satisfaction with community reintegration

One SP reported a low level of satisfaction with their PWS's rate of recovery and high level of satisfaction with community reintegration. Low satisfaction with recovery was countered by a perceived emotional shift or adjustment to the injury that had positive impacts on community reintegration.

#### Medium satisfaction with recovery and high satisfaction with community reintegration

Two SPs reported medium satisfaction with their PWS's rate of recovery and high satisfaction with rate of community reintegration. One SP reported that recovery had a direct boost in their PWS's psychological state, and the other spoke of how community reintegration exceeded expectations after reflecting back on early prognosis after injury.

#### High satisfaction with recovery and low satisfaction with community reintegration

One SP reported high satisfaction with rate of recovery and low satisfaction with rate of community reintegration and attributed their PWS's isolation from the community to working from home and COVID-19 fears.

#### High satisfaction with recovery and medium satisfaction with community reintegration

One SP attributed their high satisfaction with rate of recovery and medium satisfaction with rate of community reintegration to their perception that their PWS was not fully himself again.

### Self-efficacy and function (SCIM-III) changes over the first year after injury and perceived community reintegration

**Figure 2:**
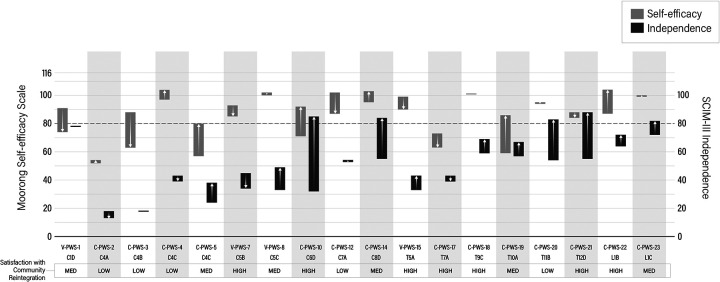
Changes in self-efficacy and independence are shown from inpatient rehabilitation to 12 mpi. Changes in self-efficacy are represented by gray bars and changes in independence are represented by black bars. Arrows represent whether there was an increase or decrease in self-efficacy and independence over the first year. Participants are ordered according to their ISNCSCI classification.

Figure 2 shows changes in self-efficacy and independence over the first year after injury and perceived satisfaction with community reintegration, and participants are ordered according to their International Standards for Neurological Classification of SCI (ISNCSCI) classification. While this study is qualitative and not organized around the quantitative Moorong SES and SCIM-III data, examining relationships of self-efficacy and independence to reported satisfaction with community reintegration may offer insights into the experiences of PWS and their SP. Seven participants experienced clinically meaningful decreases of four points or more in self-efficacy over the study. Of these individuals, three had scores below 80 at 1 year. Two individuals had scores higher than 80 at rehabilitation discharge, and one was below 80. The remaining participants who demonstrated decreased self-efficacy over the first year had scores that remained above 80. Six participants experienced a clinically meaningful increase in self-efficacy over the first year. Three participants experienced marked self-efficacy increases from inpatient rehabilitation (<80) to 1 year later (≥80). The other three started with >80. Five participants experienced non-clinically significant changes in self-efficacy. This study does not find an association between self-efficacy scores and SCI level (ISNCSCI) (Figure 2).

The association between decreasing self-efficacy and satisfaction with rate of community reintegration is unclear (Figure 2).

Regarding changes in independence as measured by the SCIM-III (Figure 2), three individuals experienced notable decreased scores of four points or more from rehabilitation to 1 year. Twelve participants experienced salient increases in scores, and three had non-significant changes in scores. As expected, higher SCIM-III scores were observed with lower levels of SCI (ISNCSCI) (Figure 2). Of the participants who experienced decreased SCIM-III scores, one reported low satisfaction with the rate of community reintegration and two reported high satisfaction. Most of the participants who experienced significant increases in SCIM-III scores reported medium to high satisfaction, while two participants reported low satisfaction in their rates of community reintegration. Of the three participants who did not experience significant changes in function, two reported low satisfaction with community reintegration, while one reported medium satisfaction. Finally, this study did not identify strong, positive trends between self-efficacy and independence.

## Discussion

This investigation of how recovery influences community reintegration during the first year after SCI, from the perspectives of individuals with SCI and their support persons, demonstrates that community reintegration is influenced by more than physical recovery. The SCI rehabilitation community's approach of maximizing strength and independence prior to discharge from inpatient rehabilitation suggests a belief that increases in physical functioning will result in greater successes in community reintegration. While functional recovery is indeed a predictor of community reintegration (39), our data reflecting nuanced personal experiences suggests that there are additional, complex influences on community reintegration. According to the ICF, participation is broadly defined as, “involvement in a life situation.” (8, p. 10). We asked study participants about their perspectives on “getting back into life” which locates the ICF domain of participation within the broader construct of community reintegration. Despite common engagement with the ICF by rehabilitation professionals, the structure of the United States market-based healthcare system introduces challenges addressing rehabilitation beyond the domains of body functions/structures, and activities, limiting direct interventions to facilitate community reintegration (3). Satisfaction with community reintegration is influenced by a complex interaction of environmental, psychosocial, physiological, and socioeconomic factors, which was revealed by the transformative nature of investigating perspectives of families living with SCI. Study participants' perspectives on how recovery, as influenced by personal and environmental factors, enlightens our understanding of conditions that shaped their experiences reintegrating into the community after SCI.

Our study is unique in that perspectives of support persons are included, as well as comparisons between Veterans and civilians. Themes related to perspectives about how recovery influences community reintegration were similar between civilian and Veteran PWS. However, civilians more frequently discussed time, or slow recovery, whereas Veterans focused on how improvements in recovery led to positive effects on community reintegration. This difference may be due to longer access to inpatient rehabilitation, of which Veterans experience an average of 2 weeks longer compared to civilian PWS in this study. Early studies on the impacts of shortening inpatient rehabilitation lengths of stay on individuals with SCI demonstrated adverse impacts on health due to secondary conditions and increased hospital readmissions (20, 21). Later studies identify lengths of stay as a complex, critical driver of healthcare costs that are influenced by healthcare system organization and processes as much as or even more so than individual patient factors (40). Other studies show that extending lengths of stay can have significant positive impacts on motor functional recovery (41, 42), and ultimately on community reintegration (39). However, individuals with SCI experience persistent, contemporary challenges seeking sufficient and appropriate rehabilitation and resources that facilitate reintegration (3, 22). One civilian discussed returning to work, however, no Veterans mentioned work in relation to recovery and reintegration. This again may be due to the benefits Veterans receive compared to civilians. Health insurance, for instance, often is tied to paid employment for civilians (23). Health insurance for Veterans, however, may be received whether or not the recipient holds paid employment. Veterans less often mentioned work when defining community reintegration (2).

Veteran and civilian SPs were in alignment on the two strongest themes, psychological/emotional state and physical or functional recovery. Hope is an undercurrent to both themes, a construct receiving renewed attention, particularly in terms of its positive association with resilience (24) and spirituality (25). Hope for positive psychological and functional outcomes may reflect something in which SPs may actively participate in and acknowledge, as a shared desire that is not always tied to resources. Further, emphasis on psychological and emotional state is in alignment with recognition of the broad psychosocial challenges experienced by the SCI community and indicates that mental health is an important concern and worthy of further exploration and consideration in healthcare policy development (26). Such policy development starts with rehabilitation providers’ knowledge and incorporation of interventions that offer effective strategies for managing psychosocial challenges.

This study revealed a moderate association between satisfaction with rate of recovery and satisfaction with rate of reintegration. While this correlation should be interpreted cautiously since the study is not powered for statistical modeling, results suggest that recovery and reintegration are indeed linked. This reifies the importance of providing PWS and SPs knowledge about opportunities for recovery as well as investigating sociostructural barriers to community reintegration. However, the outliers from the expected trajectory show that the rates of satisfaction with recovery and those of community reintegration are not always in alignment. PWS who were outliers to an expected trajectory between satisfaction with recovery and satisfaction with community reintegration demonstrated variability in other influencing variables, including functional independence and perceived self-efficacy. In one scenario, a PWS whose satisfaction with community reintegration did not keep pace with their satisfaction with recovery demonstrated impacts of external circumstances such as COVID-19 or interpersonal relationships, and presented varying changes in independence and self-efficacy. Clearly, COVID-19 posed significant barriers to reintegration and socialization (27, 28) and it is well established that personal relationships can be strained following SCI (26). However, these data demonstrate that greater independence and reasonable self-efficacy do not necessarily lead to greater satisfaction with community reintegration. By contrast, PWS whose rates of satisfaction with community reintegration transcended those of recovery experienced self-efficacy scores that indicated reasonable adjustment to SCI (19), reported themes of positivity and confidence, and benefited from family involvement and accessible housing. While they were less satisfied with their rates of recovery, almost all experienced clinically important increases in independence.

Fewer deviations from the expected trajectory were found among SPs, and most reported higher satisfaction rates with community reintegration than recovery. These SPs generally discussed having empathy, providing encouragement, and acknowledging emotional adjustment to injury, whereas SPs who were less satisfied with community reintegration focused on PWS’s isolation due to COVID-19 and not returning to preinjury statuses. These results are not surprising, as both civilian and Veteran SPs defined recovery from a psychological and emotional perspective (2) and feature prominently in how they defined community reintegration. Support persons are prudent to be concerned about their loved one's psychological health, as psychological problems of PWS have been associated with increased depression, anxiety, and burden among SPs (29, 30). Broadly speaking, when circumstances appear to be difficult for PWS, circumstances are difficult for their SPs. There is reciprocity in this relationship. When SPs have unmet needs, the health and wellbeing of PWS is at risk (31). United States health and social systems are reliant on unpaid family caregivers as Medicare and most private insurance institutions do not cover caregiving services. Further, the toll of caregiving is often underestimated. In addition to the physical demands of caregiving, the managerial, cognitive, and emotional toll that also accompanies care work is unrecognized and underappreciated (32), making the argument for increased programs and interventions that assist family caregivers.

Overall, misalignment of satisfaction with rates of recovery and satisfaction with community reintegration demonstrates an important departure from expectations that improvements in physical recovery automatically result in improvements in activity performance and participation. As we understand through the International Classification of Functioning, Disability, and Health (8), external and personal factors are influential, not the least of which include aspects of recovery from an emotional perspective. Yet, clinically important improvements in the SCIM-III and other SCI outcome measures result from statistical analyses that do not take into account the perspectives of people with lived disability experience (17). We recently examined how PWS and their SPs define recovery and community reintegration from their own perspectives and how those definitions change over time (2). The current study of how recovery influences community reintegration in combination with our investigation of definitions advances our understanding of the meaning made by PWS and SPs over the first year after injury. This transformative nature of our analysis is where we link PWS’s perspectives with existing knowledge and perspectives of researchers, with the goal of increasing precision in identifying needs and priorities of families experiencing SCI. To our knowledge, no other study has examined convergence or divergence of experienced recovery and experienced community reintegration from a qualitative perspective.

The Moorong Self-Efficacy Scale and SCIM-III surveys provided complementary data to our primary qualitative approach. These results should be interpreted with caution as the study was not powered for these measures. In looking at the entire cohort's changes in self-efficacy over the first year after injury, it is concerning that nearly half of the cohort experienced clinically meaningful decreases, particularly for the three participants whose Moorong scores were under 80, indicating lower adjustment to SCI and risk for poor long-term adjustment (19). Other factors may be at play. For example, self-efficacy has been shown to be strongly linked to elevated depressive mood (19). The present study, however, did not evaluate depression. Research demonstrates that the prevalence of depression is substantially greater after SCI compared to that of the greater population (33) and may be a contributing factor to low self-efficacy scores in our cohort. In addition, Craig et al. (34) found associations between self-efficacy, chronic pain, and chronic fatigue, factors that may have contributed to our cohort's self-efficacy scores. In terms of environmental and structural factors, it is possible that experiences navigating institutions and bureaucracy, especially during the COVID-19 pandemic, while seeking opportunities for recovery over the first year after SCI adversely affect self-efficacy and satisfaction with community reintegration (3).

Changes in independence, as measured by SCIM-III, revealed an association between greater independence and lower SCI severity, with most participants experiencing improvements in independence over the first year. Given SCIM-III has been shown to be a valid measure of functional independence (35), this result is expected. This study does not find associations between independence and self-efficacy, meaning that increases in one did not necessarily mean there were increases in the other. This finding aligns with other studies that have shown no association between self-efficacy and several measures of activity limitation (12, 36). This study does not find an association between self-efficacy scores, SCIM-III scores, and satisfaction with rate of community reintegration, suggesting that a multitude of factors influence satisfaction, including differences in how people with SCI and their families define recovery and community reintegration (2). Such heterogeneity in PWS is a contributing factor to limited evidence on effective interventions for community reintegration (5). Optimizing opportunities and reducing barriers is critical to enhancing community reintegration, and only possible by understanding the lived experience of families living with SCI. Further attention to the participation domain of the ICF and actively engaging with PWS and SPs to understand their lived experiences can lead to a personalized approach and enhanced community reintegration.

## Limitations

Notably, this study was initiated shortly before the onset of the COVID-19 pandemic, and results should be interpreted within this global context. The pandemic adversely influenced our recruitment of Veterans due to strict institutional regulations at the Veterans’ Hospital. Consequently, findings about Veterans may not be representative of the larger Veterans population, and limits our comparisons with civilians. In addition, self-efficacy and independence results should be interpreted with caution, as this study was primarily qualitative and not statistically powered for these quantitative measures. This study's patterns in self-efficacy and independence align with existing research using these measures (12, 36). Given the limited sample size for robust quantitative analysis, future studies are needed to confirm these patterns. This study did not include measures of depression, resilience, chronic pain, or fatigue, all of which potentially affect how recovery influences community reintegration. However, the open nature of qualitative inquiry offered participants the opportunity to share information if they were experiencing these phenomena. Finally, results of this work should be interpreted within the context of recovery and reintegration in the United States healthcare system.

## Conclusions

No other study has qualitatively examined convergence or divergence of experienced recovery and experienced community reintegration from the perspectives of both PWS and their SPs. Misalignment between perceived satisfaction with recovery and that of community reintegration challenges the common expectation that increased physical recovery automatically results in increased participation. Absence of a clear association between self-efficacy, functional independence, and satisfaction with community reintegration suggests that a myriad of factors contribute to perceptions of successful participation in society. Consequently, because predictions of successful community reintegration cannot be based solely on the clinical picture, it is critical to include perspectives of PWS and their support systems. Our findings lend support for representing the voices of PWS and their SPs toward the goal of successful community reintegration for all individuals after SCI.

## Data Availability

The raw data supporting the conclusions of this article will be made available by the authors, without undue reservation, upon request.
