# Supplementary material for: How recovery influences community reintegration: perspectives of persons with spinal cord injury and their support persons
Source: Front Rehabil Sci. 2025 Sep 11;6:1617764. doi: 10.3389/fresc.2025.1617764 (PMC12460264; doi:10.3389/fresc.2025.1617764)
Supplement: Supplementary file 1 [file Datasheet1.pdf]

<u>SCI Participant Interview Guide 1</u>
<u>(during initial inpatient rehabilitation stay)</u>

Identification of family member / support person to participate in interviews

Briefly describe your highest level of education and occupation. (Participation)
Briefly describe your connection to the VA/military (if Veteran).

Briefly describe your spinal injury. (Body Functions and Structures).
How did your injury happen? [ask the prompts separately, i.e. ask one, let the person answer, then ask the next]
How do you feel about your injury right now?

When you think of recovery from SCI, what does recovery mean? (Body Functions and Structures; Activity; Participation)
What are your priorities for recovery?

How would you define success at getting back into life? (Participation; Transformative)

Describe your rehabilitation experience thus far. (Body Functions and Structures; Activity).
Where have you received medical care?
What is working out the best?
What is not going well?

How have members of the rehabilitation team impacted your experiences? (Body Functions and Structures; Activity; Participation).
Who specifically has made an impact?

Describe your experiences looking for ways to affect recovery. (Participation; Transformative)
Treatments and interventions that are clinically available (describe what that means as necessary)
Experimental treatments through clinical trials (describe as necessary)
How are you finding information about resources for recovery?
Who has been helping you (if anyone)? Volunteers? Support groups?
Are you finding accessible options? (e.g., trials that are recruiting, clinically available non-experimental treatments, etc.)
Access to experts; attitudes of experts
Understandable information?

What barriers or facilitators have you run into as you have been seeking interventions to affect recovery? (Participation; Transformative).
If needed, prompt for people, institutions, knowledge, resources

Describe your thoughts on clinical trials (Body Functions and Structures; Activity; Participation; Transformative).
Interested in them?
Know how to find?

Where are you going to live after discharge from rehabilitation?
How do you feel about this? (Participation).
What needs to happen to make that successful? (Modifications, etc.)

What are your greatest concerns right now? (Body Functions and Structures; Activity; Participation; Transformative).

Is there anything you'd like to discuss that hasn't come up?

Support Person Interview Guide 1
(during loved one's initial inpatient rehab stay)

What is your relationship to (participant with SCI)? (Participation)
Length of relationship [ask the prompts separately, i.e. ask one, let the person answer, then ask the next]
How did you meet? (If not family)

What is your age;
highest level of education;
occupation? (Participation)

How do you feel about your loved one's injury? (Body Functions and Structures; Activity; Participation)
Angry, sad, hopeful, other

When you think of recovery from SCI, what does recovery mean to you with respect to your loved one? (Body Functions and Structures; Activity; Participation).
What are your priorities for recovery?

How would you define success of your loved one at getting back into life? (Participation; Transformative)

Describe your experiences as your loved one has been going through rehabilitation thus far. (Body Functions and Structures; Activity).
What is working out the best?
What is not going well?

How have members of the rehabilitation team impacted your experiences? (Body Functions and Structures; Activity; Participation).
Who specifically made an impact?

Describe your experiences looking for ways to affect recovery of your loved one. (Participation; Transformative)
Treatments and interventions that are clinically available (describe what that means as necessary)
Experimental treatments through clinical trials (describe as necessary)
How are you finding information about resources for recovery?
Who has been helping you (if anyone)? Volunteers? Support groups?
Are you finding accessible options? (e.g., trials that are recruiting, clinically available non-experimental treatments, etc.)
Access to experts; attitudes of experts
Understandable information?

What barriers or facilitators have you run into as you have been seeking interventions to affect recovery of your loved one? (Participation; Transformative).
If needed, prompt for people, institutions, knowledge, resources

Describe your thoughts on clinical trials (Body Functions and Structures; Activity; Participation; Transformative).
Interested in them?
Know how to find?

Where will your loved one be living after discharge from rehabilitation? (Participation).
How involved are you in the planning?

Describe your feelings about this.

What are your greatest concerns for your loved one right now? Body Functions and Structures; Activity; Participation; Transformative).

Is there anything you'd like to discuss that hasn't come up?

SCI Participant Interview Guide 2
(approximately 6 months' post-injury)

Last time we talked about how you define recovery. **How do you define recovery right now? What are your priorities for recovery?**

We also spoke about what successfully returning to life looks like for you. **How do you define success about "getting back into life"?** If person is overwhelmed with the COVID pandemic, try prompting with 'If you take the pandemic out of the picture for a moment, how would you define success at getting back into life'?

What other kinds of changes have occurred since we last spoke? What has gone well? What hasn't gone so well? If needed, prompt for living environment, health status, work, community participation, transportation?

How do you feel about your current living situation?

What have you done since we last spoke to affect your recovery?
If needed, prompt for therapy, other interventions or activities, clinical trials, as listed below:

Treatments and interventions that are clinically available (describe what that means as necessary)
Experimental treatments through clinical trials (describe as necessary)
How are you finding information about resources for recovery?
Who has been helping you (if anyone)? Volunteers? Support groups?
Are you finding accessible options? (e.g., trials that are recruiting, clinically available non-experimental treatments, etc.)
Access to experts; attitudes of experts
Understandable information?

Follow on question: **What barriers or facilitators have you run into as you have been seeking ways to affect recovery?**
Follow on question: **With the information that you find, how do you make decisions about what you want to do?**
Follow on question: **How have your insurance and other financial resources affected your experience accessing options for recovery?** Has your insurance changed since we last spoke?
Follow on question: **Describe how you feel about how your recovery is influencing your success at getting back to life.** Has access to interventions influenced your success?

Describe your thoughts on clinical trials. Interested in them? Know how to find?

What are your greatest concerns right now?

Where do you see yourself in 6 months?

Is there anything you'd like to discuss that hasn't come up?

<u>Support Person Interview Guide 2</u>
<u>(when loved one is about 6 months post-injury)</u>

Last time we talked about how you define recovery of your loved one. **How do you define recovery right now? What are your priorities for recovery?**

We also spoke about what successfully returning to life looks like. **How do you define success right now about your loved one "getting back into life"?** If person is overwhelmed with the COVID pandemic, try prompting with 'If you take the pandemic out of the picture for a moment, how would you define success at getting back into life'?

What other kinds of changes have occurred for you and your loved one since we last spoke? What has gone well? What hasn't gone so well? If needed, prompt for living environment, health status, work, community participation, transportation?

How do you feel about your loved one's current living situation?

What have you done since we last spoke to affect your loved one's recovery?
If needed, prompt for therapy, other interventions or activities, clinical trials, as listed below:

Treatments and interventions that are clinically available (describe what that means as necessary)
Experimental treatments through clinical trials (describe as necessary)
How are you finding information about resources for recovery?
Who has been helping you (if anyone)? Volunteers? Support groups?
Are you finding accessible options? (e.g., trials that are recruiting, clinically available non-experimental treatments, etc.)
Access to experts; attitudes of experts
Understandable information?

<u>Follow on question</u>: **What barriers or facilitators have you run into as you have been seeking ways to affect your loved one's recovery?** If needed, prompt for people, institutions, knowledge, resources
<u>Follow on question</u>: **How do you present the information that you find to your loved one?** Is she/he receptive?
<u>Follow on question:</u> **With the information that you find, how does your loved one make decisions about what she/he wants to do?**
<u>Follow on question:</u> **How have your insurance and other financial resources affected your experience accessing options for recovery of your loved one?** Has your insurance changed since we last spoke?
<u>Follow on question:</u> **Describe how you feel about how recovery is influencing your loved one's success at getting back to life.** Has access to interventions influenced his/her success?

Describe your thoughts on clinical trials. Interested in them? Know how to find?

What are your greatest concerns for your loved one right now?

Where do you see your loved one in 6 months?

Is there anything you'd like to discuss that hasn't come up?

<u>SCI Participant Interview Guide 3</u>
<u>(approximately 12 months post-injury)</u>

Last time we talked about how you define recovery
How do you define recovery right now? What are your priorities for recovery?
<u>Follow on question</u>: **How satisfied are you with your rate of recovery over this first year?**
<u>Follow on question</u>: **How complete do you feel your recovery is?** If not complete ask prompt 'What is missing from your recovery?'

We also spoke about what successfully returning to life looks like for you. **How do you define success about "getting back into life"?** If person is overwhelmed with the COVID pandemic, try prompting with 'If you take the pandemic out of the picture for a moment, how would you define success at getting back into life'?
<u>Follow on question</u>: **How satisfied are you with "getting back into life" over this first year?**
<u>Follow on question</u>: **What barriers or facilitators have you run into as you have been trying to reintegrate into the community?** If needed, prompt for people, institutions, knowledge, resources

What other kinds of changes have occurred since we last spoke? What has gone well? What hasn't gone so well? If needed, prompt for living environment, health status, work, community participation, transportation?

How do you feel about your current living situation?

What have you done since we last spoke to affect your recovery?
If needed, prompt for therapy, other interventions or activities, clinical trials, as listed below:

Treatments and interventions that are clinically available (describe what that means as necessary)
Experimental treatments through clinical trials (describe as necessary)
How are you finding information about resources for recovery?
Who has been helping you (if anyone)? Volunteers? Support groups?
Are you finding accessible options? (e.g., trials that are recruiting, clinically available non-experimental treatments, etc.)
Access to experts; attitudes of experts
Understandable information?

<u>Follow on question</u>: **What barriers or facilitators have you run into as you have been seeking ways to affect recovery?**
<u>Follow on question</u>: **With the information that you find, how do you make decisions about what you want to do?**
<u>Follow on question</u>: **How have your insurance and other financial resources affected your experience accessing options for recovery?** Has your insurance changed since we last spoke?
<u>Follow on question</u>: **Describe how you feel about how your recovery is influencing your success at getting back to life.** Has access to interventions influenced your success?
<u>Follow on question</u>: **Overall, how have your family and friends influenced your recovery?**

Describe your thoughts about clinical trials. Interested in them? Know how to find?

What is the one piece of advice you would give someone who just had a spinal cord injury?

What do you wish you knew sooner?

What are your greatest concerns right now?

How do you feel about your future?

Is there anything you'd like to discuss that hasn't come up? Since we last spoke or over the past year?
Wrap up:

- Thank you for sharing your experiences and staying with us for the whole year.
- We will be sharing the results with all the participants as they become available (sometimes there is a lag time).
- We are still here as a resource for you, including research opportunities in the future.
- You can also reach out to Kim Mackay if you need anything, even if it’s not related to research.

Support Person Interview Guide 3
(when loved one is about 12 months' post-injury)

Last time we talked about how you define recovery of your loved one.
How do you define recovery right now? What are your priorities for recovery?
Follow on question: **How satisfied are you with your loved one's rate of recovery over this first year?**
Follow on question: **How complete do you feel his or her your recovery is?** If not complete ask prompt 'What is missing from his/her recovery?'

We also spoke about what successfully returning to life looks like. **How do you define success right now about your loved one "getting back into life"?** If person is overwhelmed with the COVID pandemic, try prompting with 'If you take the pandemic out of the picture for a moment, how would you define success at getting back into life'?
Follow on question: **How satisfied are you with your loved one's "getting back into life" over this first year?**
Follow on question: **What barriers or facilitators have you run into as your loved one has been trying to reintegrate into the community?** If needed, prompt for people, institutions, knowledge, resources

What other kinds of changes have occurred for you and your loved one since we last spoke? What has gone well? What hasn't gone so well? If needed, prompt for living environment, health status, work, community participation, transportation?

How do you feel about your loved one's current living situation?

What have you done since we last spoke to affect your loved one's recovery?
If needed, prompt for therapy, other interventions or activities, clinical trials, as listed below:

Treatments and interventions that are clinically available (describe what that means as necessary)
Experimental treatments through clinical trials (describe as necessary)
How are you finding information about resources for recovery?
Who has been helping you (if anyone)? Volunteers? Support groups?
Are you finding accessible options? (e.g., trials that are recruiting, clinically available non-experimental treatments, etc.)
Access to experts; attitudes of experts
Understandable information?

Follow on question: **What barriers or facilitators have you run into as you have been seeking ways to affect your loved one's recovery?** If needed, prompt for people, institutions, knowledge, resources
Follow on question: **How do you present the information that you find to your loved one?** Is she/he receptive?
Follow on question: **With the information that you find, how does your loved one make decisions about what she/he wants to do?**
Follow on question: **How have your insurance and other financial resources affected your experience accessing options for recovery of your loved one?** Has your insurance changed since we last spoke?
Follow on question: **Describe how you feel about how recovery is influencing your loved one's success at getting back to life.** Has access to interventions influenced his/her success?
Follow on question: **Overall, how have your family and friends influenced your loved one's recovery?**

Describe your thoughts about clinical trials. Interested in them? Know how to find?

What is the one piece of advice you would give another support person of someone who just had a spinal cord injury?

What do you wish you knew sooner?

What are your greatest concerns right now for your loved one?

How do you feel about your future and your loved one's future?

Is there anything you'd like to discuss that hasn't come up?

Wrap up:

- Thank you for sharing your experiences and staying with us for the whole year.
- We will be sharing the results with all the participants as they become available (sometimes there is a lag time).
- We are still here as a resource for you, including research opportunities in the future.
- You can also reach out to Kim Mackay if you need anything, even if it's not related to research.
